# Correction to: Hippocampal subfields and their neocortical interactions during autobiographical memory

**DOI:** 10.1162/imag_x_00159

**Published:** 2024-05-20

**Authors:** Pitshaporn Leelaarporn, Marshall A. Dalton, Rüdiger Stirnberg, Tony Stöcker, Annika Spottke, Anja Schneider, Cornelia McCormick

**Affiliations:** Department of Neurodegenerative Diseases and Geriatric Psychiatry, University of Bonn Medical Center, Bonn, Germany; German Center for Neurodegenerative Diseases, Bonn, Germany; School of Psychology, The University of Sydney, Sydney, Australia; Department of Physics and Astronomy, University of Bonn, Bonn, Germany

Figure 1Ewas previously incorrect and is corrected here. No changes to the original figure legend or any other text are needed. The corrected graph inFigure 1Edisplays the comparison between the percentage of the signal change during AM and MA in hippocampal subfields along the entire longitudinal axis (DG/CA4, CA1, CA2/3, subiculum, and pre/parasubiculum), where activation during AM was significantly stronger in the pre/parasubiculum compared with the CA1, the CA2/3, and the subiculum, whereas there was a non-significant trend level in the DG/CA4.Figure 1Gis correctly presented, where the activation detected in the anterior portion of the pre/parasubiculum was found stronger in comparison to the other subfields.

**Fig. 1 (Color).**
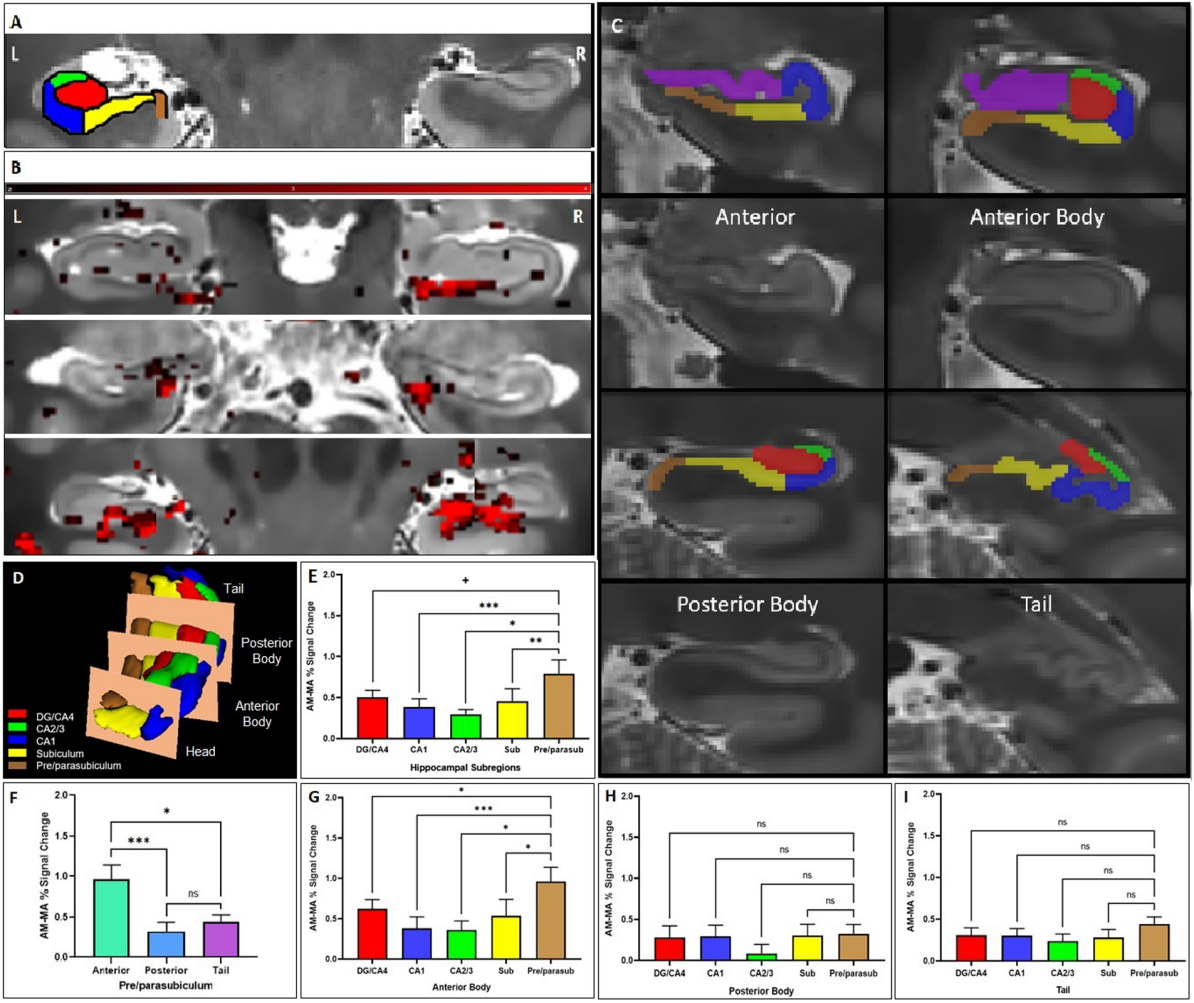
Differential hippocampal subfield engagement during AM retrieval. (A) Overlaid segmentation of labeled hippocampal subfields, including the DG, CA1-4, subiculum, and pre/parasubiculum on high-resolution structural T2-weighted scan. (B) Examples of AM versus MA activation along the longitudinal axis of the hippocampus (shown in red) from three participants (Y coordinates from upper to lower panels of 20, 17, and 27, beginning from rostral to caudal of 55 slices, respectively). (C) Examples of manual hippocampal subfields segmentation for signals extraction. The subfields along the longitudinal axis are divided into four portions of anterior, anterior body, posterior body, and tail (Y coordinates of 16, 21, 38, and 46, respectively). (D) Hippocampus subfields along the longitudinal axis. (E) The comparison between the % signal change during AM and MA in hippocampal subfields (DG/CA4, CA1, CA2/3, subiculum, and pre/parasubiculum). The pre/parasubiculum showed stronger differentiation between AM and MA than most other subfields. (F) This effect was driven by the anterior body part of the hippocampus. (G) The anterior body of the pre/parasubiculum shows greater differentiation between AM and MA than all other subfields, whereas no significant difference was found in the posterior body (H) nor the tail (I). *** p < 0.001, ** p < 0.01, and * p < 0.05, and + p < 0.1 (non-significant trend level).

