# Supplementary material for: Hippocampal subfields and their neocortical interactions during autobiographical memory
Source: Imaging Neurosci (Camb). 2024 Mar 11;2:imag-2-00105. doi: 10.1162/imag_a_00105 (PMC12247578; doi:10.1162/imag_a_00105)
Supplement: Supplementary Material [file imag_a_00105-supp.pdf]

Supplementary Materials

Figure S1.

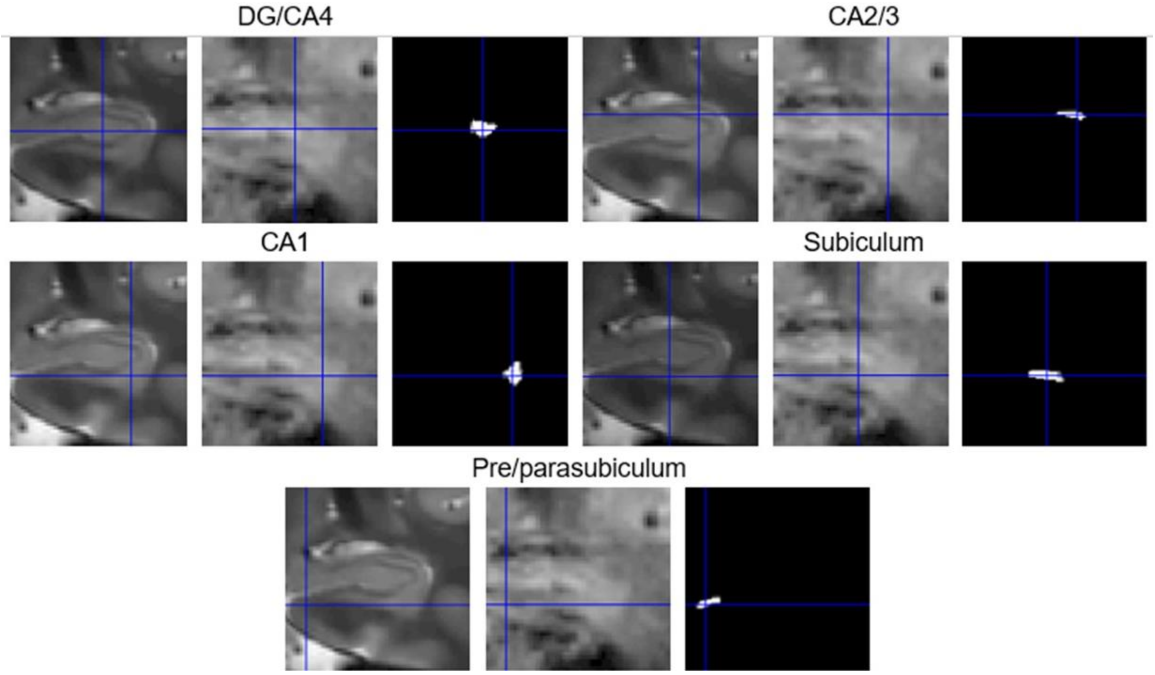

Figure S1. Examples of co-registration between structural and functional scans of five hippocampal subfields. High-resolution structural T2-weighted scans (left) are shown in alignment with their corresponding functional EPI scans (centre) and the ROI masks (right) in coronal view in the anterior body of the hippocampus. The mask of each subfield was constructed using ITK-Snap.

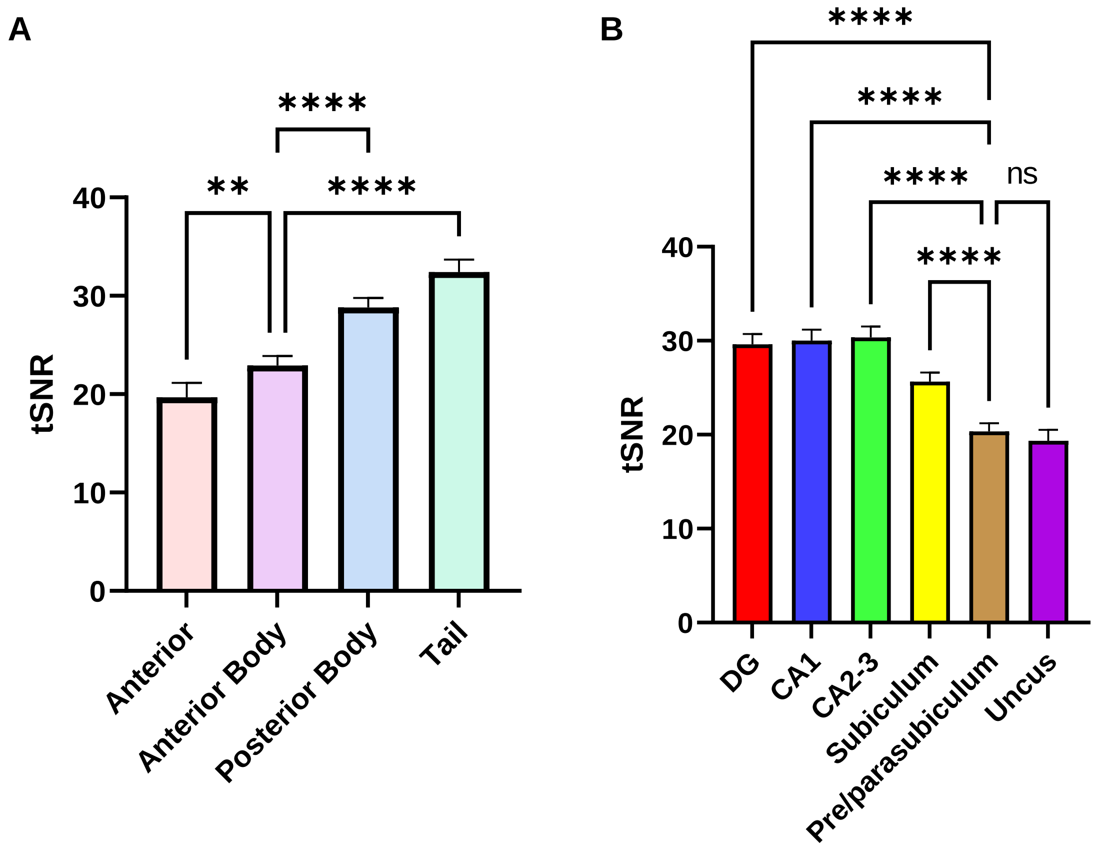

Figure

Figure S2. Comparison of the temporal signal-to-noise ratio (tSNR) across the fMRI time series along the longitudinal axis of the hippocampal subfields. A. The tSNR values ranged from 8.44 to 44.82 and multiple comparisons between the four hippocampal portions showed that the tSNR along the more anterior portions were significantly lower than the more posterior portions (F (1.722, 39.04) = 65.84, p < 0.001). Additionally, the pre/parasubiculum, as the most medial subfield, displayed lower tSNR than the other subfields (F (2.551, 58.68) = 129.2, p < 0.001). A possible explanation for this occurrence is the typical low SNR in the medial and lower regions of the brain including the hippocampus and its subfields. However, low tSNR values usually make it more difficult to detect significant differences between experimental conditions. Although we cannot rule out effects of tSNR differences on our results, low tSNR of the pre/parasubisulum was present during both experimental conditions. Therefore, despite low tSNR, we still find significant differences in this hippocampal subfield and not in the adjacent subiculum or posterior body of the pre/parasubiculum.

Table S1. Percentage of signal change during AM versus MA tasks.

	Left		*Right*		*T-Test*
	**M**	**SD**	**M**	**SD**	**P-value**
Anterior	0.8046	1.4062	0.7908	1.1998	0.8638
Anterior Body	0.9250	1.3697	0.6253	0.5293	0.1579
Posterior Body	0.4770	0.7195	0.3527	0.4562	0.1030
Tail	0.2187	0.3552	0.2164	0.5201	0.9774
DG/CA4	0.5416	0.5722	0.4036	0.3184	0.1258
CA2/3	0.4184	0.4246	0.1789	0.4936	0.1230
CA1	0.3741	0.6368	0.3381	0.4099	0.6119
Subiculum	0.4666	0.8840	0.4288	0.7922	0.4835
Pre/parasubiculum	0.7971	0.829	0.7091	0.7773	0.4313

The extracted percentages of change in activation intensity between the AM and MA in four hippocampal portions along the long axis (anterior, anterior body, posterior body, and tail) and five hippocampal subfields (DG/CA4, CA1, CA2/3, subiculum, and pre/parasubiculum), both left and right, were averaged. M= Mean, SD = Standard deviation, df = 23

Table S2. Percentage of signal change during AM versus MA tasks in the anterior body.

	Left		***Right***		***T-Test***
	**M**	**SD**	**M**	**SD**	**P-value**
DG/CA4	0.7749	0.8829	0.4734	0.5514	0.1481
CA2/3	0.5806	0.6838	0.1647	1.029	0.1564
CA1	0.4883	1.035	0.2945	0.4198	0.2181
Subiculum	0.6597	1.228	0.3989	0.8272	0.0348*
Pre/parasubiculum	0.9746	0.9099	0.9117	0.9740	0.6290

The extracted percentages of change in activation intensity between the AM and MA exclusively in the five hippocampal subfields (DG/CA4, CA1, CA2/3, subiculum, and pre/parasubiculum) of the anterior body along the longitudinal axis, both left and right, were averaged. M= Mean, SD = Standard deviation, df = 23, * $p < 0.05$

Table S3. Peak coordinates of the mean-centered PLS.

Stronger activation during AM than MA

**Region**	**side**	**MNI coordinates**			**BSR**
		**X**	**Y**	**Z**	
Frontal gyrus rectus	right	4	57	-15	9.83
Angular gyrus	left	-57	-63	20	9.65
Superior frontal gyrus	right	5	61	32	9.34
Ventromedial prefrontal cortex	left	-2	57	-14	9.02
Retrosplenial Cortex	right	11	-36	4	7.93
Retrosplenial Cortex	left	-9	-34	6	7.73
Middle occipital gyrus	left	-42	-78	15	7.46
Middle occipital gyrus	right	46	-78	15	7.34
Ventromedial prefrontal cortex	right	7	51	-9	7.18
Inferior frontal gyrus	left	-39	27	-15	6.96
Temporal pole	left	-53	-1	-29	6.86
Caudate	left	-15	18	4	6.52
Fusiform gyrus	left	-28	-35	-21	6.36
Cerebellum	right	36	-78	-41	6.33
Precuneus	left	-2	-64	33	6.02
Fusiform gyrus	right	22	-36	-20	5.82
Parahippocampal gyrus	left	-26	-16	-18	5.62
Parahippocampal gyrus	right	26	-20	-20	5.61
Cerebellum	left	-11	-80	-38	5.61
Inferior frontal gyrus	right	34	28	-15	5.56
Temporal pole	right	50	8	-24	5.29
Hippocampus	left	-27	-12	-26	4.89
Angular gyrus	right	51	-63	35	4.64
Hippocampus	right	26	-12	-24	4.45
Putamen	right	22	15	4	3.96

BSR= Boot strap ratio

Table S4. Peak coordinates of the seed PLS.

Stronger functional connectivity of the anterior body of the pre/parasubiculum during AM than MA

Region	side	MNI coordinates			BSR
		X	Y	Z	
Angular gyrus	left	-48	-58	14	13.64
Lingual gyrus	right	10	-48	-2	13.34
Angular gyrus	right	51	-57	23	13.01
Precuneus	left	-13	-47	35	9.27
Middle cingulate gyrus	right	2	9	32	9.12
Ventromedial prefrontal cortex	left	-6	40	-3	7.75
Ventromedial prefrontal cortex	right	11	40	-4	7.66
Inferior occipital cortex	left	-36	-82	-17	5.24
Fusiform gyrus	left	-28	-40	-13	5.13
Hippocampus	left	-27	-38	2	5.12
Hippocampus	right	21	-37	3	4.62
Fusiform gyrus	right	20	-36	-14	4.23
Putamen	right	20	15	4	3.23

BSR= Boot strap ratio

Table S5. Peak coordinates of the seed PLS.

Stronger functional connectivity of the anterior body of the pre/parasubiculum during MA than AM.

Region	side	MNI coordinates			BSR
		X	Y	Z	
Dorsolateral prefrontal cortex	left	-58	17	9	-9.61
Supplementary motor cortex	right	6	-17	54	-7.04
Cerebellum	right	14	-69	-46	-6.52
Ventrolateral prefrontal coretx	left	-20	54	-6	-5.84
Cerebellum	left	-13	-79	-45	-4.63
Temporal pole	left	-27	6	-23	-4.21

BSR= Boot strap ratio
